# The role of ATM and 53BP1 as predictive markers in cervical cancer

**DOI:** 10.1002/ijc.27488

**Published:** 2012-02-10

**Authors:** Frank Roossink, Hylke W Wieringa, Maartje G Noordhuis, Klaske A ten Hoor, Mirjam Kok, Lorian Slagter-Menkema, Harry Hollema, Geertruida H de Bock, Elisabeth Pras, Elisabeth GE de Vries, Steven de Jong, Ate GJ van der Zee, Ed Schuuring, G Bea A Wisman, Marcel ATM van Vugt

**Affiliations:** 1 Department of Gynaecologic Oncology, University of Groningen, University Medical Center GroningenGroningen, The Netherlands; 2 Department of Medical Oncology, University of Groningen, University Medical Center GroningenGroningen, The Netherlands; 3 Department of Otolaryngology and Head and Neck Surgery, University of Groningen, University Medical Center GroningenGroningen, The Netherlands; 4 Department of Pathology, University of Groningen, University Medical Center GroningenGroningen, The Netherlands; 5 Department of Epidemiology, University of Groningen, University Medical Center GroningenGroningen, The Netherlands; 6 Department of Radiation Oncology, University of Groningen, University Medical Center GroningenGroningen, The Netherlands

**Keywords:** cervical cancer, response to (chemo)radiation, ATM pathway, resistance, DNA damage response

## Abstract

Treatment of advanced-stage cervical cancers with (chemo)radiation causes cytotoxicity through induction of high levels of DNA damage. Tumour cells respond to DNA damage by activation of the ‘DNA damage response’ (DDR), which induces DNA repair and may counteract chemoradiation efficacy. Here, we investigated DDR components as potential therapeutic targets and verified the predictive and prognostic value of DDR activation in patients with cervical cancer treated with (chemo)radiation. In a panel of cervical cancer cell lines, inactivation of ataxia telangiectasia mutated (ATM) or its substrate p53-binding protein-1 (53BP1) clearly gave rise to cell cycle defects in response to irradiation. Concordantly, clonogenic survival analysis revealed that ATM inhibition, but not 53BP1 depletion, strongly radiosensitised cervical cancer cells. In contrast, ATM inhibition did not radiosensitise non-transformed epithelial cells or non-transformed BJ fibroblasts. Interestingly, high levels of active ATM prior to irradiation were related with increased radioresistance. To test whether active ATM in tumours prior to treatment also resulted in resistance to therapy, immunohistochemistry was performed on tumour material of patients with advanced-stage cervical cancer (*n* = 375) treated with (chemo)radiation. High levels of phosphorylated (p-)ATM [*p* = 0.006, hazard ratio (HR) = 1.817] were related to poor locoregional disease-free survival. Furthermore, high levels of p-ATM predicted shorter disease-specific survival (*p* = 0.038, HR = 1.418). The presence of phosphorylated 53BP1 was associated with p-ATM (*p* = 0.001, odds ratio = 2.206) but was not related to any clinicopathological features or survival. In conclusion, both our *in vitro* and patient-related findings indicate a protective role for ATM in response to (chemo)radiation in cervical cancer and point at ATM inhibition as a possible means to improve the efficacy of (chemo)radiation.

The standard of care for patients with advanced-stage cervical cancer has shifted over the last decade from radiotherapy alone to platinum-based chemoradiation.^1^ Despite the shift in this curative treatment modality, 5-year survival is still around 66%, which leaves ample room for improvement.^2^ Chemoradiation introduces high levels of DNA double-strand breaks (DSBs), with the aim to induce cell death.^3^, ^4^ At the molecular level, cells respond to DNA breaks with the activation of a distinct pathway called the ‘DNA damage response’ (DDR). The DDR recognises DNA damage and subsequently coordinates a cell cycle arrest with the initiation of DNA repair.^3^, ^4^ Counteracting the effects of the DDR might thus be an attractive option to improve treatment results in patients with advanced cervical cancer.

Central in the DDR is the ataxia telangiectasia-mutated (ATM) kinase. ATM plays a key role in detecting DNA DSBs and in coordinating DNA repair, cell cycle arrest and induction of apoptosis.^4^ When DNA DSBs are induced, ATM is activated through autophosphorylation on serine 1981 (Ser1981) and subsequently phosphorylates numerous downstream substrates, including cell cycle regulators, DNA repair factors and proteins involved in apoptosis.^3^, ^5^, ^6^ The importance of ATM is underscored by the observed increased radiosensitivity and cancer incidence in patients with ataxia telangiectasia (AT), bearing a mutation in the *ATM* gene.^7^ One prototypical ATM substrate is the gene product of TP53-binding protein-1 (53BP1),^8^–^11^ originally identified as a protein that binds p53.^12^ In response to DNA damage, 53BP1 is rapidly phosphorylated by ATM on multiple residues including serine 25 (Ser25) and serine 1778 (Ser1778).^6^, ^13^, ^14^ Phosphorylated 53BP1 localises to irradiation-induced foci where it promotes the activation of p53 and Chk2 and mediates the recruitment of the repair factor BRCA1.^8^–^10^ 53BP1, like ATM, is also involved in the repair of DNA breaks by promoting non-homologous end joining.^15^, ^16^ However, 53BP1 can also be detected on sites of homologous recombination and in addition influences this error-free type of repair.^17^ Altogether, these findings explain its important role in proper responses to DNA breaks, and many of the cellular defects observed in AT were recapitulated in *53BP1*^−/−^ cells, including irradiation sensitivity, growth retardation and cancer predisposition.^18^, ^19^

The aim of our study was to investigate to which degree cervical cancer cells depend on the DDR after irradiation. For this purpose, we have analysed responses of a panel of cervical cancer cell lines to ionising irradiation. We have subsequently investigated the role of ATM and 53BP1 as potential targets for radiosensitising approaches *in vitro*. Finally, we tested the predictive and prognostic properties of ATM pathway activity in tumours in a large, well-documented and consecutive series of patients with cervical cancer primarily treated with (chemo)radiation.

## Material and Methods

### Cell-line studies

The human papillomavirus (HPV)-positive cervical cancer cell lines HeLa, CaSki and SiHa (all p53 wt) as well as the HPV-negative C33A cell line (mutant p53) were cultured in DMEM:Ham's F12 (1:1), supplemented with 10% of fetal calf serum, 100 U/ml of penicillin and 100 μg/ml of streptomycin. Human embryonic 293T kidney cells, non-transformed human retinal pigment epithelial (RPE) cells and human BJ foreskin fibroblasts were cultured in DMEM, supplemented with 10% of fetal calf serum, 100 U/ml of penicillin and 100 μg/ml of streptomycin. Authenticity of cell lines was verified by DNA short-tandem repeat analysis (Baseclear, Leiden, The Netherlands). If indicated, cells were irradiated using a CIS International/IBL 637 equipped with a cesium^137^ source (0.01083 Gy/s). If indicated, cells were incubated with 10 μM of ATM inhibitor KU55399 (Tocris Biosciences, Bristol, United Kingdom).

### RNA interference

Short-hairpin RNA sequences against the human *TP53BP1* gene were previously described and validated.^20^ To produce VSV-G pseudotyped retrovirus particles, 293T cells were transfected with pRetrosuper (pRS), pRS-53BP1#1 (targeting sequence 5′-GAACGAGGAGACGGTAATA-3′) or pRS-53BP1#2 (5′-GATACTGCCTCATCACAGT-3′) and with the packaging plasmids pMDG/P and pMDG in a 3:2:1 ratio using a calcium phosphate protocol. Virus-containing supernatant culture medium was filtered (0.22 μm; Millipore, Billerica, MA), mixed with polybrene (4 μg/ml) and used for infection for three consecutive 12-hr periods. Twenty-four hours after the third infection, puromycin was added (1 μg/ml) for selection.

### Western blotting and immunofluorescence

For Western blotting, cell lysates were obtained using Mammalian Protein Extraction Reagent (Thermo Scientific, Rockford, IL), supplemented with protease inhibitor and phosphatases inhibitor cocktail (Thermo Scientific, Etten-Leur, The Netherlands). Thirty micrograms of protein was used for SDS-PAGE. Separated proteins were transferred to Polyvinylidene fluoride membranes and blocked in 5% milk in tris-buffered saline-0.01% Tween20. Immunodetection was done with antibodies directed against 53BP1 (rabbit, H-300; Santa Cruz Biotechnology, Santa Cruz, CA), MDM-2 (mouse, AB1; Merck (Calbiochem), Darmstadt, Germany), phospho-Thr68-Chk2 (rabbit, C13C1; Cell Signaling Technology, Danvers, MA), p21 (Merck (Calbiochem), Darmstadt, Germany), β-actin (mouse, A5441; Sigma-Aldrich, St. Louis, MO) and phospho-Ser1981-ATM (rabbit, EP1890Y; Epitomics, Burlingame, CA). horseradish peroxidase-conjugated antibodies (DAKO Denmark A/S, Glostrup, Denmark) were used as secondary antibodies. Visualisation was performed using Enhanced Chemiluminescence (Lumilight, Roche diagnostics, Mannheim, Germany) and a Biorad Bioluminescence device, equipped with Quantity One/Chemidoc XRS software (Biorad, Veenendaal, The Netherlands).

For immunofluorescence, cells were grown on glass cover slips. One hour after treatment, cells were fixed in 3.7% formaldehyde, blocked in 5% bovine serum albumin and stained overnight using anti-53BP1 (rabbit, H-300; Santa Cruz Biotechnology, Santa Cruz, CA) and anti-γ-H2AX (mouse, phospho-Ser139, #05-636; Millipore, Billerica, MA). Cells were counterstained with Alexa-488- and Alexa-568-conjugated secondary antibodies (Invitrogen (Molecular Probes), Eugene, OR) and 4′,6-diamidino-2-phenylindole (Sigma-Aldrich, St. Louis, MO).

### Clonogenic survival assays

Depending on the amount of irradiation, cells were seeded at 100 (0 Gy), 500 (2 Gy), 2,000 (4 Gy) or 5,000 cells per well (6 Gy) in six-well plates and allowed to adhere for 4 hr. Cells were subsequently irradiated at indicated doses. If indicated, cells were pre-treated with ATM inhibitor (KU55933, 10 μM) for 30 min. ATM inhibitor (KU55933)-treated CaSki cells were seeded up to 80,000 cells per well due to extreme irradiation sensitivity observed in initial experiments. When colony size reached an approximate minimum size of 50 cells per colony after 10–14 days, cells were fixed and stained using methanol/acetic acid/water mixture (50, 20 and 30%, respectively), containing 0.01% Coomassie brilliant blue. Surviving fraction was calculated using the plating efficiencies, using the non-irradiated controls as a reference. Results shown are averages of three independent experiments performed in triplicate.

### Apoptosis assays and proliferation measurements

Twenty-four hours after plating in six-well plates, cells were irradiated (10 Gy). If indicated, cells were pre-treated with ATM inhibitor (KU55933, 10 μM) for 30 min. Twenty-four hours after irradiation, apoptosis was assessed visually by fluorescence microscopy after staining nuclear chromatin with acridine orange. Apoptosis assays were independently performed in triplicate. To measure cell proliferation, 7,000 HeLa cells or SiHa cells were plated in 96-well plates in the presence or absence of KU55933 (10 μM). Directly after plating, or at 24, 48 or 72 hr after plating, 20 μl of 5 mg/ml 3-(4,5-dimethylthiazol-2-yl)-2,5-diphenyltetrazolium bromide (MTT) was added for 2 hr. Subsequently, culture medium was removed, and then the cells were incubated in dimethyl sulphoxide for 30 min. Absorbance was measured at 520 nm using a Biorad microplate reader. Cell growth was measured by calculating relative increases of MTT conversion. MTT conversion at Day 1 of plating was used as a reference.

### Flow cytometry

Cells were harvested at indicated time points after irradiation and fixed in ice-cold 70% ethanol. Cells were stained with rabbit anti-phospho-Ser10-Histone H3 antibody (rabbit, Cell Signaling Technology, Danvers, MA, #06570, 1:200) and subsequently stained with Alexa-488-conjugated anti-rabbit antibody (Invitrogen (Molecular Probes), Eugene, OR) and counterstained with propidium iodide/RNAse (Sigma-Aldrich, St. Louis, MO). Cell cycle distribution and phospho-Histone H3 positivity were analysed on a FACSCalibur (Becton Dickinson Biosciences, Franklin Lakes, NJ) equipped with CellQuest software. Per sample, at least 1 × 10^4^ events were analysed, and indicated results show averages and standard deviations of three independent experiments.

### Patients with cervical cancer

Immunohistochemical analysis was performed on pre-treatment tissue specimens of 375 patients with advanced-stage cervical cancer primarily treated with chemoradiation, collected between January 1980 and December 2006. Tissue specimens were used to generate a tissue microarray as described previously.^21^, ^22^ Clinicopathological data of patients, analysed in this study, are summarised in Supporting Information Table S1. The mean follow-up time was 3.99 years (range: 0.1–18.3) for all patients. For patients who were still alive at the time of their final follow-up, the median follow-up time was 6.3 years. About 189 patients (50.4%) received only radiotherapy, whereas 186 (49.6%) patients received chemoradiation. Patients who received chemoradiation were younger compared to patients who received radiotherapy alone (median 46.8 *vs*. 64.8, *p* < 0.001). All other baseline characteristics were comparable in both groups (data not shown). Immunohistochemistry was performed with antibodies against phospho-Ser1981-ATM (rabbit, EP1890Y, S1981; Epitomics, Burlingame, CA), 53BP1 (rabbit, H-300, SC-22760; Santa Cruz Biotechnology, Santa Cruz, CA), phospho-Ser25-53BP1 (rabbit, AB82559; Abcam, Cambridge, United Kingdom) and anti-γ-H2AX (mouse, #05-636; Millipore, Amsterdam, The Netherlands). As chemoradiation is associated with a better survival and is a time-dependent factor, we adjusted for treatment modality in the multivariate analyses. Additional detailed information about staining protocols, patient information, evaluation of stainings and statistical analysis can be found in the Supporting Information Document SD1.

## Results

### ATM- and 53BP1-dependent cell cycle arrest in response to irradiation

**Figure 1:**
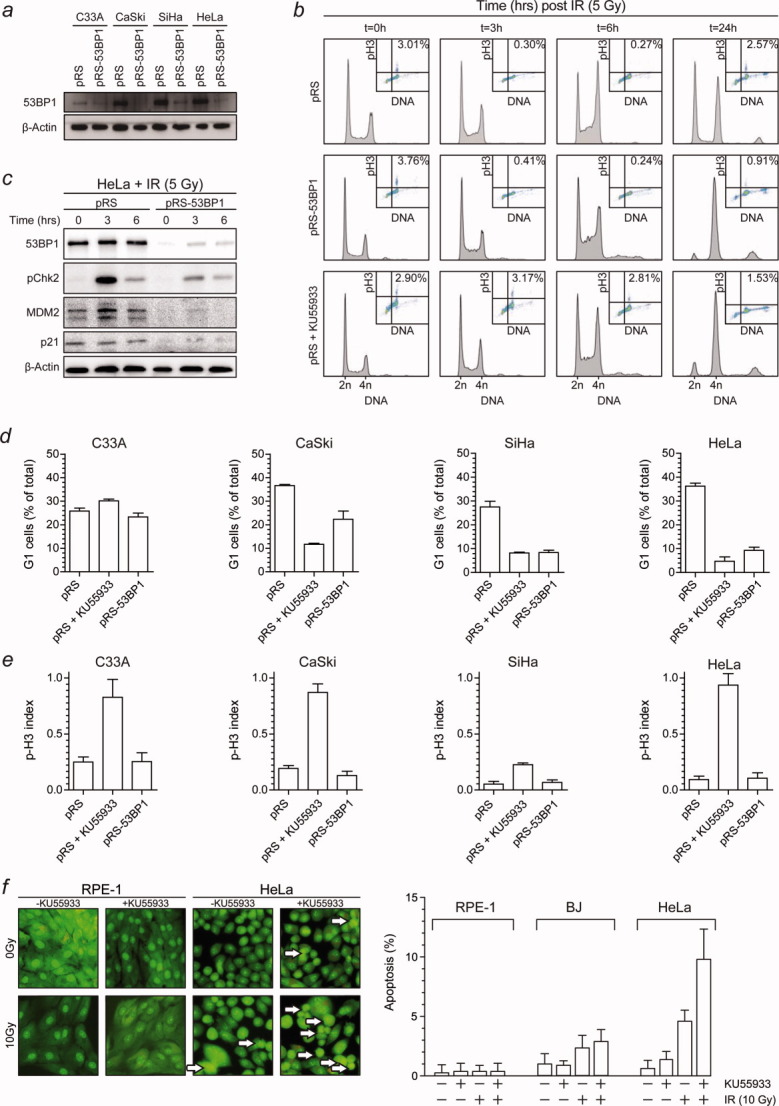
ATM- and 53BP1-dependent cell cycle arrest in irradiated cervical cancer cell lines. (*a*) C33A, CaSki, SiHa and HeLa cells were infected with pRS control virus or pRS-53BP1 shRNA virus. Whole-cell lysates of puromycin-resistant polyclonal cells were obtained and analysed with immunoblotting using indicated antibodies. (*b*) HeLa-pRS, HeLa-pRS53BP1 and HeLa-pRS cells pre-treated with KU55933 were irradiated (5 Gy) and harvested at indicated time points. Cells were fixed and stained for phospho-HistoneH3/Alexa-488 and propidium iodide/RNAse. 1 × 10^4^ events were measured by flow cytometry, and representative DNA plots are shown. Inlays show phospho-HistoneH3 stainings, and indicated percentages show phospho-HistoneH3-positive cells. (*c*) HeLa cells infected with pRS or pRS-53BP1 were irradiated (5 Gy) and harvested after indicated time periods. Immunoblotting was performed with indicated antibodies. (*d*) C33A, CaSki, SiHa and HeLa cells were treated as for Panel *b*, and relative amounts of cells with 2 N DNA content (G_1_-cells) are indicated at 24 hr after irradiation. Standard deviations of three independent experiments are shown. (*e*) C33A, CaSki, SiHa and HeLa cells were treated as for Panel *b*, and relative amounts of cells with phospho-HistoneH3-positive cells at 3 hr after irradiation are indicated. Standard deviations of three independent experiments are shown. (*f*) RPE, BJ foreskin fibroblasts and HeLa cells were treated with 10 μM KU55933 prior to irradiation. Twenty-four hours after irradiation, apoptosis was analysed by microscopic assessment of acridine orange staining. Representative images are indicated, and averages of three experiments (with at least 100 cells per experiment) are shown. Arrowheads indicate apoptotic cells. [Color figure can be viewed in the online issue, which is available at wileyonlinelibrary.com.]

We investigated the role of ATM and 53BP1 in the cellular response to irradiation in cervical cancer cells. To study cell cycle arrest in G_1_ and G_2_ (a distinct early feature of the DDR), different doses of irradiation were tested for their ability to induce cell cycle arrest and foci formation of 53BP1 and γ-H2AX (Supporting Information Figs. S1A–S1D). To subsequently study the role of ATM and 53BP1 in these responses, we used chemical inhibition of ATM (KU55933)^23^ or interfered with the expression of 53BP1 using shRNA (Fig. 1*a*) in our panel of HPV-positive (SiHa, HeLa and CaSki) cell lines and the HPV-negative C33A cell line (harbouring a p53 mutant). Cell lines stably expressing 53BP1 shRNAs as well as cell lines in which ATM was inactivated showed normal proliferation compared to their control counterparts (Fig. 1*b*). Upon irradiation (5 Gy), clear cell cycle arrests in G_1_ and G_2_ were observed (Figs. 1*b*, 1*d* and 1*e*). Interestingly, ATM inhibition or 53BP1 depletion completely ablated the G_1_ arrest in response to irradiation in all examined HPV-positive cell lines (Figs. 1*b* and 1*d*). Similar results were obtained when we used a second shRNA against 53BP1 (Supporting Information Figs. S2A–S2C). These results indicate that both ATM and 53BP1 are required for the irradiation-induced G_1_ arrest in HPV-positive cervical cancer cell lines. The p53-mutant cell line C33A did not show a significant irradiation-induced G_1_ arrest (Fig. 1*d*), which is in line with the p53-dependent G_1_ arrest after irradiation.^24^, ^25^ Indeed, neither ATM inhibition nor 53BP1 depletion further changed the proportion of G_1_ cells in C33A cells (Fig. 1*d*). We next investigated the ability of cervical cancer cells to arrest at the G_2_/M border. G_2_/M checkpoint activity was measured by the number of cells that enters mitosis after irradiation, judged by phospho-HistoneH3-positivity. Shortly after irradiation, control cells showed a clear decrease in the percentage of mitotic cells, as expected from cells with an intact G_2_/M checkpoint (Figs. 1*b* and 1*e*). Notably, ATM inhibition resulted in failure to properly arrest cells at the G_2_/M border after irradiation (Figs. 1*b* and 1*e*). In sharp contrast, cells depleted of 53BP1 still exhibited a clear decrease in mitotic cells upon irradiation comparable to that of irradiated control cells (Figs. 1*b* and 1*e*). These results indicate that ATM, but not 53BP1, is required for a proper G_2_/M arrest in response to irradiation, and furthermore, these results show that the G_1_ and the G_2_/M arrests have different molecular requirements in cervical cancer cells.

Although previous studies have attributed an irradiation induced G1-arrest to p53 function,^26^ we still observed a prominent G1-arrest in all HPV-positive cervical cancer cell lines, despite the compromised p53 function in these cells. Therefore, we tested p53 function and observed expression of p53-target genes MDM2 and p21 in HeLa cells, albeit at low levels (Fig. 1*c*). Interestingly, depletion of 53BP1 resulted in a virtually complete loss of expression of MDM2 and p21, suggesting that the residual activity of p53 in cervical cancer cells depends on 53BP1 (Fig. 1*c*).

Regarding ATM inhibition as a therapeutic option, we compared the ability of ATM inhibition to induce apoptosis in HeLa cells *versus* human non-transformed RPE cells and human BJ foreskin fibroblasts. Although ATM inhibition clearly elevated the levels of apoptotic HeLa cells after irradiation, non-transformed RPE cells or BJ foreskin fibroblasts did not show any significant elevation of apoptosis levels in response to combined treatment with KU55933 and irradiation (Fig. 1*f* and Supporting Information Fig. S1E).

### ATM, but not 53BP1, is required for clonogenic survival after irradiation

**Figure 2:**
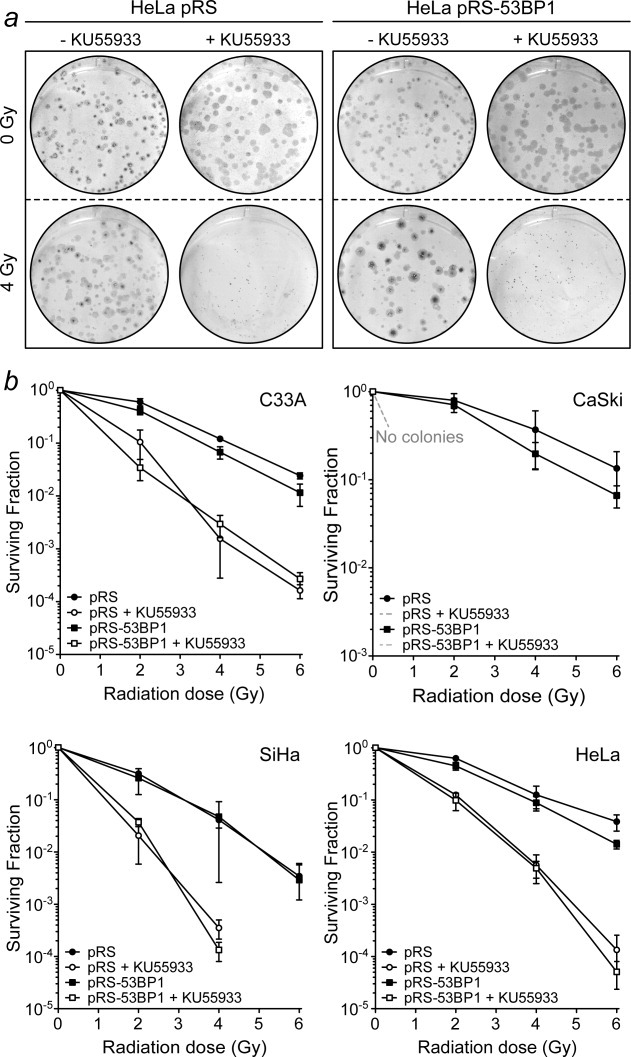
ATM- and 53BP1-dependent clonogenic survival in irradiated cervical cancer cell lines. (*a*) C33A, CaSki, SiHa and HeLa cells were infected with pRS control virus or pRS-53BP1 shRNA virus. Cells were plated in six-well plates and subsequently irradiated with indicated amounts of ionising irradiation and allowed to grow colonies. If indicated, cells were treated with KU55933 prior to irradiation. Surviving colonies were stained. (*b*) Quantification of colony numbers. If no colonies survived, a dashed line is shown. Data are shown from three independent experiments.

Our cell cycle analysis showed that ATM inhibition or loss of 53BP1 clearly led to different defects in response to irradiation (Fig. 1). Reduced ability to initiate cell cycle arrest in response to irradiation may also translate into altered survival kinetics of irradiated cells. To test this, we subsequently analysed clonogenic survival. To exclude that ATM inhibition resulted in altered growth rates and thereby influenced the results of the clonogenic survival assay upon irradiation, MTT proliferation assays were conducted and revealed that ATM inhibition on its own did not significantly alter growth rates of HeLa cells or SiHa cells (data not shown; Supporting Information Fig. S3A). Importantly, inhibition of ATM resulted in a dramatic reduction in clonogenic survival after irradiation, observed in all cell lines tested (Figs. 2*a* and 2*b*). Notably, CaSki cells appeared much more sensitive to ATM inhibition compared to other cell lines (Fig. 2*b*). In contrast, loss of 53BP1 only resulted in a very moderate loss of clonogenic survival after irradiation in all four cell lines (Figs. 2*a* and 2*b*). As expected, loss of 53BP1 did not further sensitise cells that were treated with ATM inhibitor (Figs. 2*a* and 2*b*).

Altogether, our results indicate a differential requirement for ATM or 53BP1 with respect to irradiation-induced cell cycle arrest and show that ATM inhibition dramatically radiosensitises cervical cancer cells as judged by survival assays. These results imply that the levels of ATM activity, perhaps even prior to irradiation, may determine the effect of (chemo)radiation.

### Predictive value of ATM and 53BP1 for response to treatment

**Figure 3:**
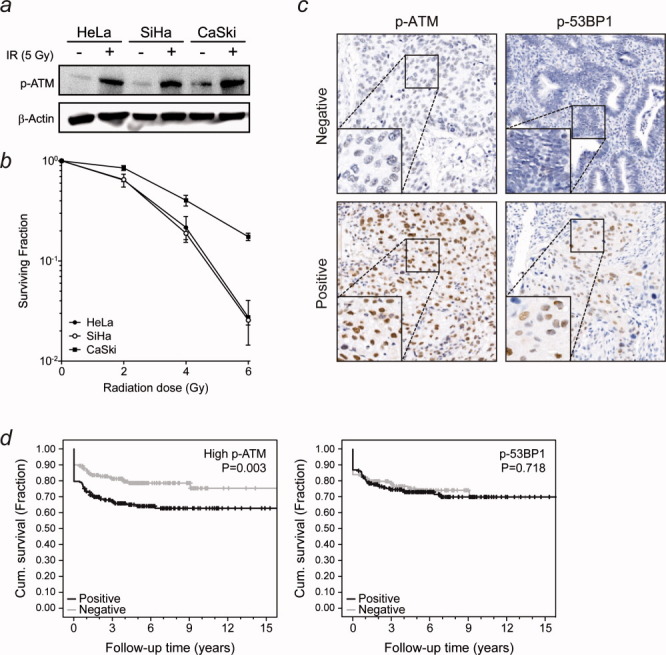
Relationship of ATM and 53BP1 expression to locoregional disease-free survival. (*a*) HeLa, SiHa and CaSki cells were left untreated or irradiated (5 Gy). Thirty minutes after irradiation, cells were lysed and immunoblotted for phospho-Ser1981-ATM and β-actin. (*b*) HeLa, SiHa and CaSki cells were plated in six-well plates and subsequently irradiated with indicated amounts of ionising irradiation and allowed to grow colonies. Surviving colonies were stained, and average colony numbers of three experiments are indicated. (*c*) Representative immunostaining for p-ATM and p-53BP1 in advanced-stage cervical cancer is shown. (*d*) Kaplan-Meier plots of locoregional disease-free survival related to the expression of p-ATM and p-53BP1. [Color figure can be viewed in the online issue, which is available at wileyonlinelibrary.com.]

To investigate the predictive value of ATM activity for response to irradiation in cervical cancer cells, we analysed the levels of phospho-Ser1981-ATM before and after irradiation (Fig. 3*a*). HeLa and SiHa cells have very low baseline levels of phospho-ATM, in contrast to CaSki cells that have activated ATM even prior to irradiation (Fig. 3*a*). Interestingly, baseline amounts of phospho-ATM seem to correlate with radioresistance, as CaSki cells were significantly more resistant to irradiation than HeLa and SiHa cells as observed in clonogenic survival assays (Fig. 3*b*). These findings suggest that ATM activity prior to treatment can be used as a predictor of response to irradiation. To test the specificity of antibodies in paraffin-embedded material, we analysed paraffin-embedded cervical cancer cell lines irradiated in the presence or absence of ATM inhibitors (Supporting Information Fig. S4A). Our results showed that irradiation clearly increases phospho-S1981-ATM levels as well as phospho-S25-53BP1 levels, a process that is completely reverted after pre-treatment with ATM inhibitor KU55933 (Supporting Information Fig. S4A). These findings indicate that phospho-S1981-ATM and phospho-S25-53BP1 can be detected specifically in paraffin-embedded cervical cancer cells and that both stainings reflect ATM activity.

To investigate whether indeed ATM activity has predictive value for response to (chemo)radiation, we examined phospho-Ser1981-ATM [phosphorylated ATM (p-ATM)] levels in pre-treatment cervical cancer tissues. Representative p-ATM stainings in cervical cancer tissue are shown in Figure 3*c* (details of staining evaluation are described in Supporting Information Document SD1). Any positive nuclear staining (≥10% of intensity ≥1) for p-ATM was observed in 344 of 349 patients (98.6%), indicating that ATM is activated at least to some degree in virtually all patients. High levels of p-ATM expression, however, were observed only in 183 patients (52.4%).

We analysed the expression levels of the ATM substrate 53BP1. Positive nuclear expression for 53BP1 was observed in 100% of the tumours with similar intensity, and thus, no statistical analysis for 53BP1 expression could be performed. Positive nuclear phospho-S25-53BP1 [phosphorylated 53BP1 (p-53BP1)] expression, representing ATM activity, was observed in 180 of 311 tumours (57.9%). As expected for a direct substrate of ATM,^6^, ^10^, ^13^ a positive signal for p-53BP1 was more frequently found in tumours with high p-ATM in comparison to negative/low p-ATM [odds ratio (OR) = 2.206; 95% CI = 1.383–3.519; *p* = 0.001].

We evaluated the relationship between clinicopathological data *versus* p-ATM and p-53BP1 expression (Table 1). Logistic regression analysis showed higher expression levels of p-ATM in advanced stage (≥IIb) tumours (OR = 1.851; *p* = 0.007). In addition, tumour diameter (≥4 cm; OR = 1.848; *p* = 0.014) and age (OR = 1.005: *p* = 0.006) were also related to p-ATM expression. None of the clinicopathological features showed a statistical relationship with positivity for p-53BP1 (Table 1).

**Table 1:** Relationship between tumour staining for p-ATM and p-53BP1 *versus* clinicopathological data

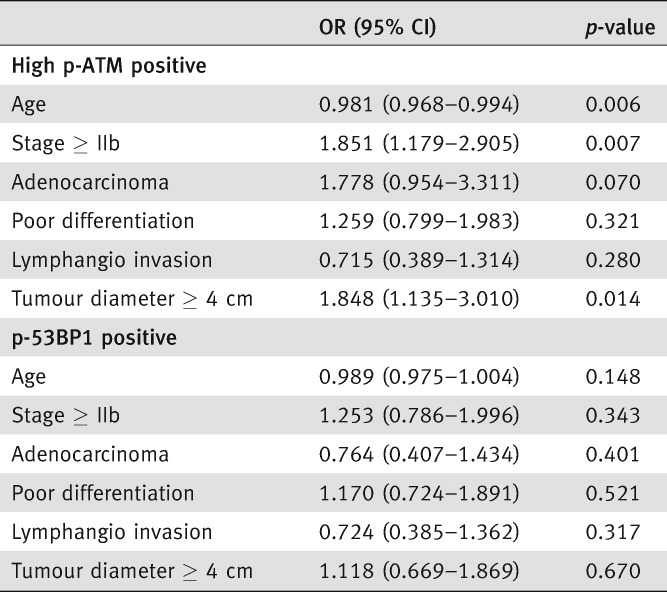

### p-ATM and p-53BP1 in relation to response to (chemo)radiation and survival

To analyse the relationship of p-ATM and p-53BP1 protein expression with response to (chemo)radiotherapy, two models were used as described previously.^22^ In Model I, wherein treatment response is based on locoregional disease-free survival, 364 patients (97.1%) could be analysed. The evaluation of locoregional disease-free survival was used as it is a relevant measurement of local effects induced by (chemo)radiation, which induces ATM pathway activation. In this model, high p-ATM was related to poor locoregional disease-free survival in univariate Cox regression analysis [hazard ratio (HR) = 1.817; *p* = 0.006] as well as in multivariate analysis (HR = 1.650; *p* = 0.022), whereas p-53BP1 expression was not related (Table 2). Figure 3*d* depicts locoregional disease-free survival in relation to high p-ATM and p-53BP1 expression. In this analyses, the log-rank *p*-value for high p-ATM expression was *p* = 0.003. To further strengthen our hypothesis that high levels of p-ATM are associated to the response to (chemo)radiotherapy, we evaluated response-to-treatment in a second model. In Model II, we separated our data in two subsets of patients with the highest contrast in treatment response (see Patients and Methods section in Supporting Information Document SD1).^22^ In this model, a statistical significant association between high p-ATM and poor response to treatment was found in univariate logistic regression analysis (OR = 2.567; *p* = 0.011) as well as in multivariate analysis (OR = 2.336; *p* = 0.039) (Table 2). p-53BP1 was not related to response to (chemo)radiation. After selection of these extreme groups, the relationship of p-ATM with poor response-to-treatment was even stronger compared to Model I. These data indicate that pre-treatment ATM activity levels, but not phosphorylation status of 53BP1, is relevant for the response to (chemo)radiation in patients with cervical cancer.

**Table 2:** Immunostaining in relation to poor response to therapy

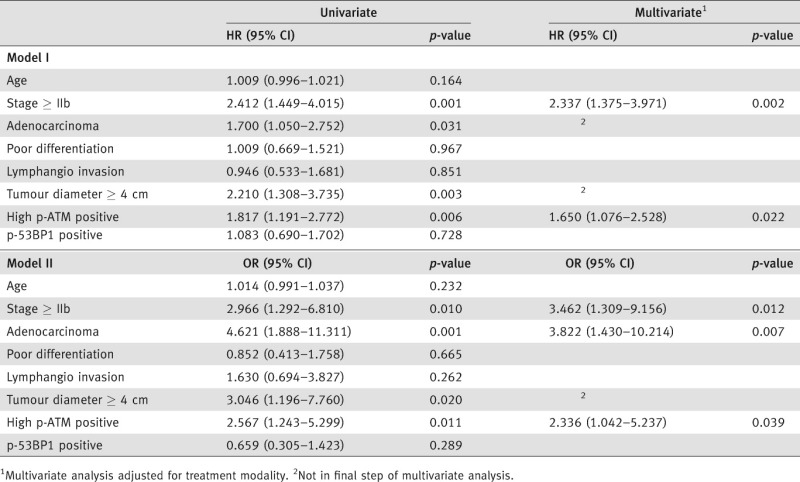

Finally, we analysed the expression of p-ATM and p-53BP1 in relation to disease-specific survival. During follow-up, 201 of 375 201/375 = 54%) patients died. In 155/201 = 77% of these patients, death was related to cervical cancer (disease-specific survival). In line with our results described above, no relationships were observed between p-53BP1 expression and disease-specific survival. However, we found that high p-ATM expression was related to worse disease-specific survival (HR = 1.418; *p* = 0.038) in univariate analysis (Table 3), again underscoring a role for ATM activity in cervical cancer behaviour.

**Table 3:** Immunostaining of p-ATM and p53BP1 in relation to disease-specific survival

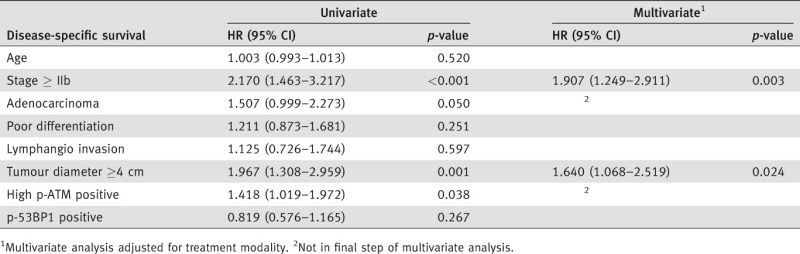

## Discussion

Our study shows a critical role for active ATM in the response of cervical cancer to irradiation, both in cervical cancer cell lines as well as in patients with cervical cancer treated with (chemo)radiation. Our *in vitro* studies indicate that cervical cancer cells, even in the presence of HPV E6 expression, which blocks p53 function, still undergo a robust G_1_ cell cycle arrest in response to irradiation. HPV-positive cervical cancer cells require both ATM activity and the presence of 53BP1 for induction of this irradiation-induced G_1_ arrest. In addition, ATM, but not 53BP1, is required for induction of a G_2_/M cell cycle arrest.

The requirements for ATM and 53BP1 for induction of an irradiation-induced G_1_ arrest are likely explained by residual p53 function in HPV-positive cervical cancer cells, which most often carry wild-type alleles of p53.^27^ Both ATM and 53BP1 have been described to regulate p53 function. Although ATM has been extensively described to directly regulate p53,^4^ a role for 53BP1 in the regulation of p53 is controversial. 53BP1 was initially demonstrated to bind p53 and promote p53 activity^12^, ^28^; however, T-cells from 53BP1^−/−^ mice still showed robust irradiation-induced p53 responses.^29^ Our results indicate that 53BP1 is required for p53 function, albeit in a background with compromised p53 levels. However, in HPV-positive cervical cancer cells this does not appear to have functional consequences, since 53BP1-depletion did not dramatically alter clonogenic survival of cell lines after irradiation. Moreover, 53BP1-phosphorylation was not associated with response to (chemo)radiation.

In our study, the inhibition of ATM in cervical cancer cells interfered with the irradiation-induced G_1_ and G_2_/M cell cycle arrest, in line with the loss of checkpoint function in cells from patients with AT.^4^, ^7^, ^30^ ATM inhibition furthermore severely decreased clonogenic survival for all tested cervical cancer cell lines, indicating that cervical cancer cells heavily depend on the ATM signalling axis for survival after irradiation. Interestingly, when ATM was inhibited in irradiated non-transformed RPE cells or BJ foreskin fibroblasts, no induction of apoptosis was observed, in contrast to the apoptotic effects of ATM inhibition on HeLa cells, implying that a therapeutic window for ATM inhibition may be present. Further research, however, is required to investigate the long-term toxicity profile of ATM inhibition in normal cells. In addition, investigation of the combined effects of radiotherapy and ATM inhibition in normal tissues are needed to further establish ATM as a therapeutic target for radiosensitisation.

These observations, as well as our findings that high baseline levels of active ATM correlate to increased clonogenic survival after irradiation, suggest that high levels of activated ATM levels are beneficial for cervical tumour cell survival. Previously, it has been acknowledged that the ability to repair therapy-induced DNA damage counteracts the efficacy of therapy in a number of tumour types. For instance, high expression levels of the repair enzyme MGMT counteracts the effects of alkylating agents and predicts poor prognosis in gliomas.^31^ Conversely, low expression level of the DNA damage repair gene ERCC1 correlates with prolonged survival of patients with non-small cell lung cancer.^32^

To analyse whether activation status of ATM was also related to therapy outcome, we assessed the expression of p-ATM and the expression of p-53BP1. To our knowledge, this is the first time that ATM and 53BP1 have been assessed by immunohistochemistry in their activated phosphorylated state in a large, well-documented and consecutive series of patients with cervical cancer primarily treated with (chemo)radiation. Our data suggest that high expression levels of p-ATM are related to poor locoregional disease-free survival. Currently, only very limited data concerning ATM and 53BP1 expression in relation to response to (chemo)radiation and survival in other malignancies is present. The few studies that investigated ATM expression show an ambiguous picture. In patients with pancreatic cancer, ATM expression in their tumours was not a prognostic factor.^33^ In contrast, expression of ATM in colorectal cancer was related to good survival in a large series of patients, although only a subset was treated with genotoxic therapy.^34^ In oesophageal cancer and early stage breast cancer, ATM expression did not predict response to therapy.^35^, ^36^

Our results show that expression of p-ATM was related to response-to-treatment, whereas expression of p-53BP1 was not related. Importantly, our clinical data were in line with our cell line data, in which we show that 53BP1 inhibition did not affect clonogenic survival upon irradiation. Although predictive roles for 53BP1 were unknown in cervical cancer, high expression levels of 53BP1 were associated with poor responses to cisplatin-based therapy in lung cancer.^37^ In analogy, 53BP1 was overexpressed specifically in those ovarian tumours that showed resistance to paclitaxel/carboplatin-based therapy.^38^ In addition, 53BP1 was frequently lost in hereditary breast cancers, where it was suggested to relieve the genomic instability caused by BRCA1 loss.^17^ The diversity of these results could be explained by differences in treatment modality between and even within these studies. Moreover, our study focussed predominantly on the activation status of both proteins, rather than expression levels only. Finally, carcinogenesis of cervical cancer is fundamentally different from that of the tumour types under study in the latter reports. It may very well be that early inactivation of p53 through HPV infection account for differences in prognostic factors, especially those functioning within the DDR.

ATM is activated in response to chemoradiation. The relatively high levels of p-ATM that we observed in our therapy-naive cervical tumour specimens could represent continuous activation of the ATM-regulated DDR as a result of deregulated proliferation, as also reported for other tumour types.^39^, ^40^ Alternatively, elevated levels of p-ATM prior to (chemo)radiation may point at DNA damage-independent functions of ATM, such as the recently reported role for ATM in sensing oxidative stress levels.^41^

In summary, on the basis of our results, high phospho-ATM levels is an independent predictor for poor response in cervical cancer. In addition, targeting ATM kinase activity could be an interesting therapeutic option to sensitise tumour cells for (chemo)radiation in patients with cervical cancer who have high tumour levels of active ATM before start of (chemo)radiotherapy. Our data, in line with other reports, show that inhibition of ATM by targeted drug application results in enhanced sensitivity to radiotherapy,^23^, ^42^, ^43^ recapitulating the radiosensitivity phenotypes of cells of patients with AT.^4^, ^7^, ^30^, ^44^ Moreover, in short-term *in vitro* assays, a therapeutic window for ATM inhibition appears to be present for ATM inhibition when the toxicity to non-cancer cells is taken into account. This differential sensitivity to ATM inhibition may be related to HPV-dependent rewiring of the cell cycle machinery. Other investigations have reported enhanced radiosensitivity by inhibition of ATM in several malignancies.^45^–^49^ Although ATM inhibitors are still in pre-clinical development, our study suggests relevance of ATM-targeted agents and warrants a further assessment of ATM inhibition as a (chemo)radiosensitising treatment in patients with advanced-stage cervical cancer. However, besides increased radiosensitivity, patients with AT also show increased cancer development,^4^, ^7^, ^44^ and therefore, prolonged ATM inhibition should be avoided. In clinical settings, only a scenario in which short-term ATM inhibition can be combined with the local induction of DNA damage seems feasible. In this respect, it is very relevant that reversible ATM inhibitors have recently been described and that transient ATM inhibition was shown to reach radiosensitising effects in cancer cells.^47^

## Abbreviations

AT: ataxia telangiectasia
ATM: ataxia telangiectasia mutated
DDR: DNA damage response
DSB: double-strand break
HPV: human papillomavirus
HR: hazard ratio
OR: odds ratio
p-ATM: phosphorylated ATM
p-53BP1: phosphorylated 53BP1
53BP1: p53-binding protein-1
RPE: retinal pigment epithelium
Ser: serine
Thr: threonine
