# Supplementary material for: The role of ATM and 53BP1 as predictive markers in cervical cancer
Source: Int J Cancer. 2012 Feb 10;131(9):2056–66. doi: 10.1002/ijc.27488 (PMC3504092; doi:10.1002/ijc.27488)
Supplement: Supplementary file 5 [file ijc0131-2056-SD5.doc]

**Supplemental Document S1**

**Immunofluorescence**

Cells were grown on glass cover slips and at the indicated time points after radiation, coverslips were removed from the culture dish and washed twice with PBS. Cells were then fixed for 10 minutes in 3.7% paraformaldehyde at room temperature and subsequently washed with PBS. Cells were permeabilised using PBS, containing 0.1% Triton-X100 for 5 minutes and subsequently washed extensively. Cells were stained with primary mouse antibodies against γ-H2AX (Upstate, pS139, #05-636, 1:200) or rabbit antibodies against 53BP1 (Santa Cruz Biotechnology, B-300, 1:200) in PBS-0.05% Tween20, supplemented with 5% BSA for 3 hours at room temperature. After extensive washing, cells were incubated with anti-mouse or anti-rabbit secondary antibodies, conjugated with Alexa-488 and Alexa-568 respectively and were counterstained using DAPI. Images were obtained on a Leica DMRXA fluorescence microscope and a minimum of 20 nuclei were scored for the presence of γ-H2AX or 53BP1 foci per condition.

**Patients**

Our group has established a large database of paraffin-embedded formalin-fixed tumour material and follow-up data from cervical cancer patients treated at the University Medical Center Groningen (UMCG). Routine patient follow-up time was at least 5 years or until September 2010. Staging of patients was according to FIGO guidelines. This patient cohort and corresponding treatments, review board approval and tissue microarray (TMA) construction has been previously described (1).

**Evaluation of response to (chemo)radiation**

Approximately 8-10 weeks after completion of (chemo)radiation either hysterectomy or biopsy was performed if a patient was (technically) classified as operable. Therefore, not all patients in our database underwent a post-treatment biopsy and/or hysterectomy. As a consequence, the response to (chemo)radiation could not be evaluated based on this parameter in all patients. Therefore, we evaluated response to (chemo)radiation retrospectively in two ways. First (Model I), the response to (chemo)radiation was determined based on loco-regional disease-free survival. Loco-regional disease-free survival is defined as the period from diagnosis until loco-regional progression of disease during treatment or loco-regional recurrence. Patients were excluded from the analysis, if the location of the recurrence was unknown. Second (Model II), response to (chemo)radiation was determined in two subsets of patients, with supposedly the largest difference in treatment response. In the first subset, patients with no residual tumour-material in their post treatment specimen and without loco-regional recurrence during follow-up with a minimum of 2 years were selected. The second subset consists of patients with clinical evidence of disease progression during treatment or clinical evidence of disease persistence at examination after completion of primary treatment. Both models also have been described and used previously (1).

**Immunohistochemistry**

Tissue microarray (TMA) construction has been previously described (1). TMAs were immunohistochemically stained with monoclonal antibodies against p-ATM (1:50; Rabbit IgG, Epitomics, Clone EP1890Y, S1981) exclusively detects the phosphorylated (Ser1981) ATM product (2). Total levels of 53BP1 were stained using a rabbit polyclonal antibody against 53BP1 (53BP1, 1:1000; 16h; Rabbit IgG, Santa Cruz Biotechnology, clone H-300; SC-22760). Phosphorylated 53BP1 was detected using polyclonal anti-phospho-Ser25-53BP1 (1:25, 16h; Rabbit IgG, Abcam, Clone AB82559) and polyclonal anti-phospho-Ser1778-53BP1 (1:50, 16h; Rabbit IgG, Cell Signaling, Clone 2675). Ser139 phosphorylated H2AX was detected using monoclonal anti-γ-H2AX (1:200, Mouse IgG, Millipore, #05-636).

From our TMAs, 3 µm sections were cut and placed on amino-propyl-etoxy-silan (APES) coated glass slides. Slides were subsequently deparaffinised using xylene and rehydrated using a multistep process from ethanol to phosphate buffered saline (PBS). Antigen retrieval for the p-ATM antibody was done using microwave treatment in EDTA buffer (pH=8). For 53BP1 and p53BP1 stainings, antigen retrieval was performed microwave treatment in citrate buffer (pH=6). Endogenous peroxidase activity was blocked by incubating with 0.3% hydrogen peroxidase for 30 minutes. All antibodies were detected using rabbit anti-goat/biotin labelled secondary antibodies and horse-radish-peroxidase (HRP) conjugated streptavidin, except for γ-H2AX, where rabbit anti-mouse/HRP and goat anti-rabbit/HRP were used. Counterstaining was achieved using hematoxylin. All incubations with primary antibodies were performed at room temperature for 1 hour. Anti-γ-H2AX, anti-53BP1 and anti-phospho-53BP1 stainings were detected using 3’3-diaminobenzidinetetrahydrochloride (DAB). Anti-p-ATM staining was visualised using EnVision (DAKO). Counterstaining was achieved using hematoxylin.

**Antibody validation for immunohistochemistry**

In order to validate our antibodies for immunohistochemistry, we used paraffin-embedded HeLa or MCF-7 cell pellets that were left untreated or were radiated with 5 Gy irradiation. 53BP1 was present in untreated and irradiated cells (data not shown). To analyze the phosphorylation-status of 53BP1, we used antibodies that recognize two previously validated ATM phosphorylation sites on 53BP1, Ser25 and Ser1778 (3-4). Phospho-Ser25-53BP1 and phospho-Ser1778-53BP1 hardly showed reactivity in non-irradiated cells, but showed massive reactivity in irradiated paraffin-embedded cells, in line with 53BP1 being phosphorylated on both these sites by ATM in response to irradiation (Supplemental Figure S2A and data not shown). Surprisingly, the anti-phospho-Ser1778-53BP1 showed extensive cytoplasmic staining (data not shown) and these results indicated that the phospho-Ser1778 antibody may also stain a non-specific protein in paraffin-embedded material and our further analyses of 53BP1 phosphorylation were therefore restricted to phospho-Ser25-53BP1 (referred to as p-53BP1).

In order to confirm that ATM is responsible for phosphorylation of Ser25 on 53BP1 in response to irradiation, we analysed paraffin-embedded HeLa cell pellets, and included cells that were incubated with ATM inhibitor KU55933 prior to irradiation (Suppl. Fig. S2A). Reassuringly, ATM inhibition completely ablated the phosphorylation of 53BP1 (Suppl. Fig. S2A) as well as phosphorylation of the established ATM substrate H2AX on Ser139 under the same conditions (Suppl. Fig. S2B). We next examined the phosphorylation status of ATM itself. To this end, we used the extensively characterised ATM autophosphorylation site Ser1981 (5) (Suppl. Fig. S2B). Already in the absence of irradiation-induced DNA damage, HeLa cells showed high levels of Ser1981-phosphorylated ATM (Suppl. Fig. S2B), which were further induced after irradiation (Fig. S2B). As expected, reactivity of phospho-Ser1981-ATM (referred to as p-ATM) was virtually absent when cells were pretreated with the ATM inhibitor KU-55933 (Suppl. Fig. S2B).

**Evaluation of immunohistochemistry**

Staining, intensities were semi-quantitatively scored as negative (0), weak positive (1), positive (2), and strong positive (3). In addition, the percentage of positive tumour cells per staining intensity was documented for each core, since not only the level of expression of either p-ATM or (p-)53BP1, but also the proportion of cells that are positive could play an important role in the response to chemoradiation.

We have classified p-ATM immunostaining data into ‘*high’* expression, which is defined as patients with positive nuclear immunostaining with an intensity of at least 2 present in at least 75% of tumour cells. For p-53BP1 staining, we have classified into the following three groups: *'Negative'*, which is defined as all patients with negative (0) staining or patients with weak staining intensity (1) in 0-25% of tumour cells, *'Positive'*, which is defined as patients with a staining intensity of 1 in more than 25% of tumour cells, or patients with a staining intensity of 2 or 3. TMA evaluation was performed independently by two observers without prior knowledge of the clinical data. A concordance of more than 90% was found between both observers, for all immunostainings. Subsequent evaluation of disconcordant cases was performed to reach a consensus score. Only patients with at least two evaluable tumour cores were included for statistical analysis.

**Statistical analysis**

Statistical analysis was performed using SPSS 16.0 for Windows (SPSS Inc., Chicago, IL). The Student’s t-test was used to analyse differences in age. Other baseline characteristics were compared with the Pearson’s χ2 test. In addition, logistic regression models were used to evaluate positive staining and clinicopathological characteristics, with immunostaining being the dependent factors and the clinicopathological characteristics being the independent factors. Logistic regression was also used to evaluate associations between immunostainings of p-53BP1 and p-ATM. To identify factors involved in response to (chemo)radiation, response to (chemo)radiation (dependent) was evaluated in relation to clinicopathological factors and immunostaining (independent) using Cox-regression analysis for Model I and logistic regression analysis in Model II. Disease-specific survival was defined as the time from diagnosis until the last follow-up alive, death due to other causes than cervical cancer or death due to cervical cancer. Survival was visualised using the Kaplan-Meier method and Mantel-Cox log rank test was used to evaluate the differences between these curves. Disease-specific survival was analysed using the Cox regression analysis. Since (chemo)radiation is a time-dependent factor and associated with a better survival, multivariate analyses were adjusted for treatment modality. Variables with a *P-*value of <0.10 in univariate analysis were excluded stepwise in multivariate analysis; in the final step, only factors with a *P-*value of <0.05 were included. *P-*values <0.05 were considered statistically significant.

**References:**

1. Noordhuis MG, Eijsink JJ, Ten Hoor KA, Roossink F, Hollema H, Arts HJ, et al. Expression of epidermal growth factor receptor (EGFR) and activated EGFR predict poor response to (chemo)radiation and survival in cervical cancer. Clin Cancer Res. 2009;15:7389-97.

2. Bartkova J, Horejsi Z, Koed K, Kramer A, Tort F, Zieger K, et al. DNA damage response as a candidate anti-cancer barrier in early human tumorigenesis. Nature. 2005;434:864-70.

3. Jowsey P, Morrice NA, Hastie CJ, McLauchlan H, Toth R, Rouse J. Characterisation of the sites of DNA damage-induced 53BP1 phosphorylation catalysed by ATM and ATR. DNA Repair (Amst). 2007;6:1536-44.

4. Matsuoka S, Ballif BA, Smogorzewska A, McDonald ER, 3rd, Hurov KE, Luo J, et al. ATM and ATR substrate analysis reveals extensive protein networks responsive to DNA damage. Science. 2007;316:1160-6.

5. Bakkenist CJ, Kastan MB. DNA damage activates ATM through intermolecular autophosphorylation and dimer dissociation. Nature. 2003;421:499-506.
