# Supplementary material for: The role of ATM and 53BP1 as predictive markers in cervical cancer
Source: Int J Cancer. 2012 Feb 10;131(9):2056–66. doi: 10.1002/ijc.27488 (PMC3504092; doi:10.1002/ijc.27488)
Supplement: Supplementary file 6 [file ijc0131-2056-SD6.doc]

**Supplemental Figure 1: DNA damage responses in cervical cancer cell lines**

**A**-**C,** HeLa cells were left untreated or were irradiated with indicated doses of IR. Twenty-four hours after irradiation, cells were harvested, fixed and stained using propidium iodide/RNAse. In panel **A**, the average amount of G_1_ cells (containing 2N DNA) is indicated. In panel **B**, the average amounts of S-phase cells are indicated. Data represent averages and standard deviations of three independent experiments. **C**, HeLa cells were fixed at one hour after irradiation (5 Gy). Cells were permeabilised and stained for γ-H2AX-Alexa-568 and 53BP1-Alexa-488. Representative cells are indicated and inlays represent magnified images of nuclear areas. **D**, C33A, CaSki, SiHa and HeLa cells were treated as for panel C. Amounts of γ-H2AX and 53BP1 foci per nucleus were counted and averages and standard deviations of at least 20 cells per condition are indicated. **E.** BJ foreskin fibroblasts were left untreated or treated with KU55933 for 30 minutes prior to irradiation (10 Gy). Twenty-four hours after irradiation. Representative images are indicated.

**Supplemental Figure 2: DNA damage responses after 53BP1-depletion.**

**A**. HeLa cells were infected with pRS control virus, pRS-53BP1#1 or pRS-53BP1#2 shRNA virus. Whole cell lysates of puromycin-resistant polyclonal cells were obtained and analysed with immunoblotting using indicated antibodies. **B**, HeLa-pRS, HeLa-pRS53BP1#1 and HeLa-pRS53BP1#2 were left untreated or were irradiated (5 Gy) and harvested after 24 hours. Cells were fixed, stained with propidium iodide/RNAse. 1*10^4^ events were measured by flow cytometry and representative DNA plots are shown. **C**, Quantification of the results obtained for panel B. Amounts of G_1_ cells from three independent experiments were quantified and averages and standard deviations are indicated.

**Supplemental Figure 3:**

**A**. HeLa cells were plated at 7000 cells per well in 96-wells plates in the presence or absence of KU55933. After 24, 48 or 72 hours, cell growth was measured using MTT conversion. Shown averages and standard deviations are from six replicates and growth was related to MTT conversion levels at t=0h.

**Supplemental Figure 4: Validation of antibodies for immunohistochemistry.**

HeLa cells were left untreated or were irradiated (5 Gy). Alternatively HeLa cells treated with KU55933 for 30 minutes prior to irradiation. Thirty minutes after irradiation, cells were trypsinised and incorporated in solidifying agarose to yield blocks containing HeLa cells. Agarose blocks were further processed for fixation in formalin and embedded in paraffin blocks, using identical conductions as are used in diagnostic pathology. Paraffin-embedded cells were sliced, de-paraffinised, stained for γ-H2AX, phospho-Ser-1981-ATM or phospho-Ser25-53BP1, and counter-stained with hematoxylin.
