# Supplementary material for: The role of ATM and 53BP1 as predictive markers in cervical cancer
Source: Int J Cancer. 2012 Feb 10;131(9):2056–66. doi: 10.1002/ijc.27488 (PMC3504092; doi:10.1002/ijc.27488)
Supplement: Supplementary file 7 [file ijc0131-2056-SD7.doc]

Supplemental Table S1: Patient and tumour characteristics

|  |  | **n=375** | | |  |
| --- | --- | --- | --- | --- | --- |
|  | **Age at diagnosis** |  |  |  |  |
|  | Median | 54 |  |  |  |
|  | Range | 20 | - | 91 |  |
|  |  |  |  |  |  |
|  | **FIGO stage** | **N** |  | **%** |  |
|  | Ib1 | 42 |  | 11% |  |
|  | Ib2 | 27 |  | 7% |  |
|  | IIa | 51 |  | 14% |  |
|  | IIb | 179 |  | 48% |  |
|  | IIa | 11 |  | 3% |  |
|  | IIIb | 51 |  | 14% |  |
|  | IVa | 14 |  | 4% |  |
|  |  |  |  |  |  |
|  | **Histology** |  |  |  |  |
|  | Squamous | 311 |  | 83% |  |
|  | Adenocarcinoma | 52 |  | 14% |  |
|  | Other | 12 |  | 3% |  |
|  |  |  |  |  |  |
|  | **Differentiation grade** |  |  |  |  |
|  | Good/moderate | 223 |  | 64% |  |
|  | Poor/undifferentiated | 128 |  | 36% |  |
|  | Unknown | 24 |  |  |  |
|  |  |  |  |  |  |
|  | **Lymph vascular space involvement** |  |  |  |  |
|  | No | 248 |  | 82% |  |
|  | Yes | 54 |  | 18% |  |
|  | Unknown | 73 |  |  |  |
|  |  |  |  |  |  |
|  | **Tumour volume** |  |  |  |  |
|  | 0-4 cm | 99 |  | 29% |  |
|  | ≥4 cm | 238 |  | 71% |  |
|  | Unknown | 38 |  |  |  |
|  |  |  |  |  |  |
|  | **Follow-up duration (years)** |  |  |  |  |
|  | Median | 3.99 |  |  |  |
|  | Range | 0.13 | - | 18.31 |  |

Abbreviation: FIGO, International Federation of Gynaecologists and Obstetricians.
